# Diseases of the Kidneys

**Published:** 1905-06-10

**Authors:** 


### PROGRESS IN MEDICINE AND SURGERY.

### DISEASES OF THE KIDNEYS.

**Treatment of Bright's Disease.**—In acute Bright's disease Bradford [1] is of opinion that the milk diet, which is ordinarily stated to be three pints a day, may be restricted to a pint or a pint and a half. This diminishes the work of the kidneys considerably. Further, if elimination of the kidneys is defective, excretion is best obtained through the bowel rather than through the skin. Purgation eliminates water, some of the solid constituents of the urine, and intestinal products. In chronic Bright's disease it is, generally, not advisable to make a mere diminution in the amount of albumen the main object of treatment; a diet may lead to an increase in the albuminuria and yet be more beneficial to the patient than one under which the daily loss of albumen is slightly less. A milk diet, by promoting diuresis, may diminish the percentage amount of albumen though the daily loss remains constant. Albuminuria is very refractory to treatment, and neither dietetic measures nor drugs produce any very obvious direct effects. This is well seen in even the more trivial forms of albuminuria—so-called physiological albuminuria—where restriction of diet produces but small results, whilst rest produces great ones. Many cases often spoken of as chronic Bright's disease are in reality cases of albuminuria dependent on former damage inflicted on the kidney and are not progressive at all. These patients often present but few signs apart from the albuminuria. There is some damage to the renal filter allowing the proteids of the blood to pass through, but no progressive disease. Far more reliable information is to be obtained by a study of the quantity of urine and its specific gravity. Most destructive lesions of the kidney lead to an increase in the quantity, provided that the lesion is not one causing dropsy. A copious flow of urine, especially if it be of low specific gravity, is to be regarded as a sign of the impairment of the physiological entirety of the organ. The presence of dropsy influences treatment. Since dropsical fluids contain from 10 to 20 times as much urea per cent. as the amount normally in the blood, and probably other substances as well which are toxic in their action, it is advisable, as far as possible, to remove them by puncture or drainage rather than by purgation or diuresis, for by the latter mode of elimination these waste products must pass through the blood. It is probable that, in many forms of renal disease, and especially in granular kidney, a moderate degree of cardiac hypertrophy is, on the whole, beneficial. Although such patients run the risk of cerebral hæmorrhage when the pressure is excessive, yet it is easier to treat such cases than those in which cardiac dilatation takes place. Cases of chronic Bright's disease vary a good deal in the degree of cachexia and of anæmia; some are pale and cachectic, whilst others look well, though with well-marked physical signs of progressive disease. Both experimental and clinical observations show that extensive destruction of the renal substance, though involving grave disturbance of nutrition, is not necessarily accompanied by uræmia, which is a toxic condition, due to some substance elaborated in the body owing to the disturbed metabolism resulting from the renal lesion. The actual onset of uræmia may possibly be brought on by a sudden diminution in the excretory activity of the kidney. The diet of a patient with chronic Bright's disease should contain but small quantities of proteid. The exact kind of proteid should be determined by its digestibility. So-called white meats and fish have no special virtue in diminishing the work of the kidney. Purgation should be by salines, though the occasional administration of a small dose of calomel is beneficial rather than prejudicial. The sleeplessness of renal disease is largely dependent on high tension, and nitro-glycerin or erythrol tetranitrate should be administered; nitrite of sodium is rarely of use owing to the severe gastric symptoms often set up. When high tension is more extreme a moderate venesection is frequently of great value; but free purgation has much more lasting effects. Chloral is useful owing to its producing a marked lowering of the blood pressure. Where blood pressure is low owing to cardiac dilatation, absolute rest in bed and the cautious administration of the infusion of digitalis are indicated. The cachexia of renal disease requires to be treated by food, and harm may be done by keeping the patient on a low diet. A most useful drug in this condition is arsenic. Pilocarpin and hot-air baths are of little value in treating uræmia. Venesection is of great value, and very striking improvement may be seen as a result of the combination of venesection and transfusion. Morphia is often very useful in this condition, especially in the cases where epileptiform seizures are present, and there is no embarrassment of the respiration owing to dropsical or inflammatory effusions.

**Salt in Bright's Disease.**—Widal[2] has made the discovery that in certain cases of Bright's disease dropsy can be produced at will by feeding the patient with salt. In a case of Bright's disease salt was alternately given in excess (four times), and in deficit (five times). Each time the salt was in excess the body weight was increased by hydration and twice palpable dropsy was developed. With a deficit of salt the body weight diminished from loss of water. Normally we take in and excrete 15 grams of salt, and healthy kidneys will excrete as much as 30 grams. Ordinary milk diet will give 5½ grams of salt. This may in disease be below or above the kidney excretory level. If above, the dropsy remains. In such a case, when saltless bread and meat and potatoes are substituted for milk there is relief to the dropsy. Milk diet is thus not usually necessary; if no extra salt is used in cooking or at meals a solid diet may actually prove better.

[1] Clin. Jour., Feb. 22, 1905. [2] Med. Chron., December, 1904.

*(To be continued.)*

